# Leveraging consumer behavior insights and information-based interventions to mitigate food waste in restaurant environments: a step toward sustainable food systems management

**DOI:** 10.3389/fnut.2026.1755198

**Published:** 2026-03-26

**Authors:** Manze Li, Tianwei Xie, Hanxiang Luo, Zhaoyang Xiang

**Affiliations:** 1 College of Economics and Management, Huazhong Agricultural University, Wuhan, China; 2 Dongshin University, Naju, Republic of Korea

**Keywords:** crowding, food sustainability, food waste, information processing fluency, information-based intervention

## Abstract

**Background:**

A rapid increase in food waste has gained global attention, and providing consumers with sustainability information in restaurants is considered an important anti-food waste intervention. However, there is ongoing debate regarding its effectiveness. This study explores whether crowding—a common dining situation in restaurants—has a potential impact on the effectiveness of information-based interventions.

**Methods:**

Three experiments were conducted with a total sample of 1,124 participants (*N*_total_ = 1,124) to examine the relationship between restaurant crowding levels and the effectiveness of different types of information-based interventions.

**Results:**

The results indicate a matching effect between crowding and information-based intervention types. Specifically, under high crowding conditions, prompt-type interventions (vs. feedback-based interventions) significantly increase consumers’ willingness to pack leftovers and reduce over-ordering behaviors. In contrast, under low crowding conditions, feedback-based interventions (vs. prompt-type interventions) are more effective in mitigating food waste. Furthermore, information processing fluency mediates this matching effect.

**Discussion:**

These findings reveal how environmental factors in restaurants influence the effectiveness of information-based interventions and provide practical guidance for marketers to select appropriate information-based intervention types based on the level of restaurant crowding to effectively prevent food waste.

## 1 Introduction

Food waste has gained widespread attention worldwide. According to statistics, global food waste reaches as high as 1.05 billion tons each year, with a value exceeding 1 trillion USD, and the production of this food requires one-third of the world’s agricultural land (1). In summary, food waste not only poses a serious challenge to global food security (2) but also means that a large amount of agricultural resources is wasted (3). In terms of the sources of food waste, most food waste results from consumers’ unreasonable consumption practices (4). Therefore, to curb food waste worldwide, the United Nations commits to reducing global per capita consumer food waste by 50% by 2030 [Sustainable Development Goal [SDG] 12; (1)]. Given that the food waste rate in consumer dining (11.7%) is much higher than that in households (4.8%) (5), reducing consumer food waste in restaurants has become a critical issue to address to achieve the Sustainable Development Goal (SDG) (6).

Currently, stakeholders have developed and implemented various policies and activities to curb consumer food waste in restaurants. Among these, information-based interventions have become one of the most common approaches (7). Compared to rewards and punishments (8) or changes in tableware design (9), delivering anti-food waste information to consumers is characterized by simplicity in implementation and low costs (10). Therefore, many countries encourage the use of information-based interventions in restaurants. For example, in the UK, restaurants are recommended to place informational prompts on menus, posters, buffet areas, or table cards to guide consumers in reducing plate waste (11). In China, the Anti-Food Waste Law explicitly requires all restaurants to display anti-food waste cards to remind consumers to reduce food waste (12).

However, as food sustainability information becomes more prevalent in restaurants, the evaluation results of information-based interventions remain contentious. Most scholars acknowledge the positive role of information-based interventions in mitigating consumer food waste. For example, Stöckli et al. (7) successfully reduced consumer food waste by placing informational cards on the tables of a pizza restaurant; Gong et al. (13) conducted a field experiment in a university cafeteria in China and demonstrated that both prompt-type and feedback-based information are effective in reducing food waste; and Jiang et al. (14) conducted a randomized controlled trial in a restaurant, and the results showed that information-based interventions prevented approximately 10% of food from being wasted. However, a few studies argue that the impact of information-based interventions on food waste is relatively weak. For example, Yu et al. (15) conducted a survey in restaurants in Beijing and found that placing informational cards had a very limited effect on reducing consumer food waste; Visschers et al. (16) conducted a field experiment in two university cafeterias in Switzerland and found that information-based interventions did not reduce plate waste. On the one hand, the inconsistent conclusions spark discussions about which type of information-based intervention is more effective (14), and on the other hand, this divergence suggests the presence of unidentified moderating factors in the impact of information-based interventions on consumer food waste (17).

Based on a review of the literature, we found that the majority of studies on the effectiveness of anti-food waste information focus on the interaction between intervention materials and consumer behavior. They often overlook the potential role of environmental factors. However, scholars in consumer behavior have emphasized that environmental cues in consumption settings can strongly influence consumer behavior (18). Prior research showed that dining out has become increasingly frequent in the majority of countries worldwide (19). As a result, crowding has become one of the most common environmental factors faced by consumers in restaurant settings (20). Some studies have begun to recognize that crowding affects consumers’ available cognitive resources. For example, Consiglio et al. (21) argued that high crowding creates excessive stimuli and distractions. This can reduce attention and deplete cognitive resources. Jin et al. (22) provided further support using eye-tracking methods. They found that crowded environments reduce visual attention to target products. In addition, Tan et al. (23) showed that crowding increases self-control failure. This effect is often explained by reduced capacity for deliberate thinking caused by cognitive resource depletion (20). Information-based interventions rely on consumers’ information processing to influence behavior, and information processing is a cognitive activity that consumes cognitive resources (17). It is therefore reasonable to expect that restaurant crowding may shape the effectiveness of anti-food waste information. Unfortunately, this issue has received little attention in existing research. This gap may help explain why prior studies report mixed findings on the effects of information-based interventions.

To address the research gap, this study conducted a field experiment and two laboratory experiments to examine the following research questions. (1) Does restaurant crowding influence the effectiveness of information-based interventions in reducing consumer food waste? (2) If such an effect exists, which type of information-based intervention performs better under different crowding levels, and through what psychological mechanism? By addressing these questions, this study develops a matching model between the environmental factors and information-based intervention types at the theoretical level. The model shows that the optimal type of information-based intervention differs across levels of crowding. It also confirms that information processing fluency is the key psychological mechanism underlying this matching effect. These findings help bridge existing debates on which type of information intervention is more effective. They also deepen understanding of how information-based interventions influence consumer food waste and highlight the potential positive role of crowding in consumption settings. On a practical level, these findings highlight the importance for marketers to consider the potential impact of restaurant crowding on the effectiveness of information-based interventions and offer practical guidance on how to select the appropriate information-based types based on crowding levels to reduce consumer food waste effectively.

## 2 Theory and hypothesis development

Information-based intervention refers to delivering information to the target group to encourage them to take desired actions (24). Typically, the types of information-based interventions used in anti-food waste campaigns can be categorized into antecedent interventions or consequence interventions (14). Antecedent interventions alter the context that precedes the target behavior, while consequence interventions encourage behavior change by highlighting the consequences of the target behavior (7). As described by Whitehair et al. (10), we primarily explore the effectiveness of two common types of information-based interventions in anti-food waste campaigns. The first type is prompt-type intervention, which uses clear and straightforward language to directly suggest waste reduction to consumers (25), making it one of the most prominent antecedent interventions (14). The second type is feedback-based intervention, which presents numerical information related to food waste and aims to raise consumers’ awareness of saving food (26), making it one of the most prominent consequence interventions (14). Although these two types of information-based interventions have received wide attention, their effectiveness in reducing food waste has not reached a clear conclusion. Whitehair et al. (10) found that prompt-type interventions reduced consumer food waste by 15%, while feedback-based interventions showed no significant effect. In contrast, Liao et al. (25) argued that prompt-type and feedback-based interventions were equally effective in reducing food waste. This view was supported by Gong et al. (13). However, Xu et al. (26) reported the opposite pattern. They found that feedback-based interventions were significantly more effective than prompt-type interventions in reducing consumer food waste. These mixed findings suggest the need to introduce new theoretical perspectives to further explain and refine the mechanisms through which information-based interventions influence consumer food waste.

The effectiveness of information-based interventions depends on whether the information is sufficiently persuasive to consumers. Processing fluency theory is one of the most widely used frameworks in social psychology for explaining how information influences attitude change. It provides an important basis for evaluating information persuasiveness: when individuals experience higher processing fluency, they are more likely to act on the information (27). This occurs for two main reasons. First, people tend to view information that is easy to process as more credible. As a result, they are more likely to rely on it when making decisions (28). Second, high processing fluency gives information a pleasant quality and produces positive affect. This positive feeling increases the likelihood that individuals will follow the information (29). Conceptually, information processing fluency refers to an individual’s subjective experience of how fluently information is processed. It is related to, yet distinct from, clarity, credibility, and information quality. On the one hand, high processing fluency often leads individuals to perceive information as clear and credible (28). On the other hand, clarity, credibility, and information quality are more often viewed as characteristics of the information itself. They are closely associated with information design. For example, Ganz and Grimes (30) argued that information credibility depends on the information source and message content. Wang and Li (31) found that adding more arguments to a message can significantly improve perceived information quality. Dass et al. (32) noted that clarity, as a design element of a message, can be controlled through word choice. In contrast, information processing fluency emphasizes individuals’ subjective perceptions. It depends on the interaction between individual characteristics and information characteristics. Processing fluency theory further suggests that matching effects are one of the most important sources of processing fluency (33). Specifically, when an individual’s information processing route matches the characteristics of the information, the individual experiences higher processing fluency during information processing (34).

Whitehair et al. (10) were among the first to propose a possible matching effect in information-based interventions. Drawing on the Elaboration Likelihood Model (ELM), they suggested that people use two distinct information processing routes: the peripheral route and the central route. The peripheral route relies on intuition and requires little logical reasoning (35). It is therefore more suitable for processing prompt-type information. In contrast, the central route involves more deliberate thinking and logical evaluation (35). It is more suitable for processing feedback-based information. However, an important issue is often overlooked. Individuals tend to choose how to process information based on their available cognitive resources (17). This implies that the information processing route that individuals adopt may not always match the type of information-based intervention they receive (36).

Prior research showed that crowding, as a common environmental factor in consumption settings (23), can influence consumers’ cognitive resources (21). For example, based on dual-processing theory, Hock and Bagchi (20) argued that under low crowding conditions, individuals have sufficient cognitive resources. As a result, they are more likely to rely on a deliberative, cognitive thought-based route when making decisions. In contrast, high crowding limits cognitive resources. This condition leads individuals to rely more on automatic affective reactions. As the ELM largely overlaps with dual-processing theory, we can integrate the arguments of Whitehair et al. (10) with the findings of Hock and Bagchi (20) and, on this basis, develop the following predictions: In dining environments with low crowding, consumers are more likely to use the central route to process information. In this case, feedback-based interventions lead to higher information processing fluency and make consumers more likely to take actions to reduce food waste. In contrast, in dining environments with high crowding, consumers tend to rely on the peripheral route and make quick and intuitive judgments. In this case, prompt-type interventions lead to higher information processing fluency and make consumers more likely to take actions to reduce food waste. Consequently, we propose the following:

*H1a*: In low crowding dining situations, feedback-based interventions (vs. prompt-type interventions) are more effective in mitigating consumer food waste.

*H1b*: In high crowding dining situations, prompt-type interventions (vs. feedback-based interventions) are more effective in mitigating consumer food waste.

*H2*: Information processing fluency mediates the matching effect between crowding and information-based intervention types on consumer food waste in restaurants.

### 2.1 Overview of studies

To test whether the hypothesis holds, we conducted three experiments to ensure that the findings satisfy both internal validity and external validity. Study 1 was a field experiment that received permission from the restaurant. The aim was to verify whether there was a matching effect between restaurant crowding levels and the information intervention types. Study 2 was a laboratory experiment that replicated the findings of Study 1 by activating participants’ perceived crowding through image priming. It also tested whether information processing fluency mediated the matching effect and ruled out a potential alternative explanation. In Study 3, we changed the stimuli and directly measured participants’ food waste behavior (over-ordering), further validating the previous findings and enhancing the robustness of the conclusions. All studies were approved by the Scientific Ethics Committee of Huazhong Agricultural University (HZAUHU-2025-0102).

### 2.2 Study 1

Study 1 was a field experiment conducted in a Chinese restaurant. We evaluated the levels of crowding during different time periods, designed prompt-type and feedback-based intervention cards, and placed them on the restaurant tables. Subsequently, we weighed the leftover food of the consumers. The goal was to examine whether there were differences in consumer food waste at varying crowding levels and to preliminarily test whether there was a matching effect between crowding and information-based intervention types.

### 2.3 Pretest 1

We needed to ensure the information-based intervention cards met the experimental requirements. Based on the intervention materials designed by Whitehair et al. (10), we adapted the intervention information from the China Central Television’s (CCTV) anti-food waste campaign. We recruited 70 participants (51.4% of female; *M*_age_ = 31.8, *SD* = 9.43) through the Credamo platform (one of the leading online survey platforms in China) and randomly showed participants either a feedback-based intervention card or a prompt-type intervention card.

**Figure 1:**
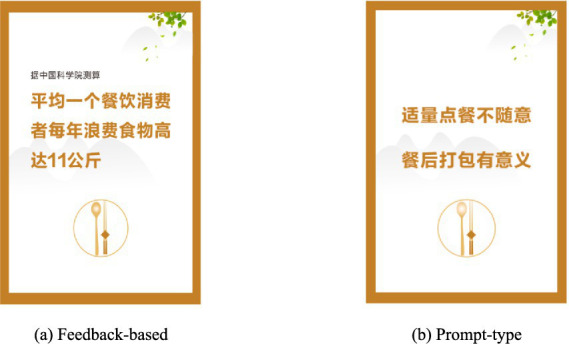
Information-based intervention cards in study 1. **(a)** Feedback-based: On average, each consumer wastes 11 kg of food per year in restaurants. **(b)** Prompt-type: Order appropriately, packing leftovers makes sense.

As shown in Figure 1, the feedback-based intervention group received a card that read, “On average, each consumer wastes 11 kg of food per year in restaurants”, while the prompt-type intervention group received a card that read, “Order appropriately, packing leftovers makes sense”. Afterward, both groups were informed about the definitions of the information-based intervention types and were asked to evaluate the card type (“I think this card belongs to a prompt-type/feedback-based intervention, 1 = strongly disagree, 7 = strongly agree”) and the esthetic appeal (“How visually appealing do you think the card’s design is? 1 = very unattractive, 7 = very attractive”).

We found that participants in the feedback-based intervention group significantly believed that the card they viewed belonged to a feedback-based intervention (*M*_feedback_ = 5.97, *SD =* 1.18; *M*_prompt_ = 2.89, *SD* = 1.76; *F* (1, 68) = 74.29, *p* < 0.001). Likewise, participants in the prompt-type intervention group significantly believed that the card they viewed belonged to a prompt-type intervention (*M*_feedback_ = 2.29, *SD =* 1.20; *M*_prompt_ = 5.26, *SD* = 1.50; *F* (1, 68) = 83.50, *p* < 0.001). The difference between the two groups in terms of the esthetic appeal of the card was not significant (*M*_feedback_ = 5.46, *SD* = 0.92; *M*_prompt_ = 5.51, *SD* = 1.10; *F* (1, 68) = 0.06, *p* = 0.814). These results indicated that the experimental materials met the requirements.

### 2.4 Pretest 2

**Figure 2:**
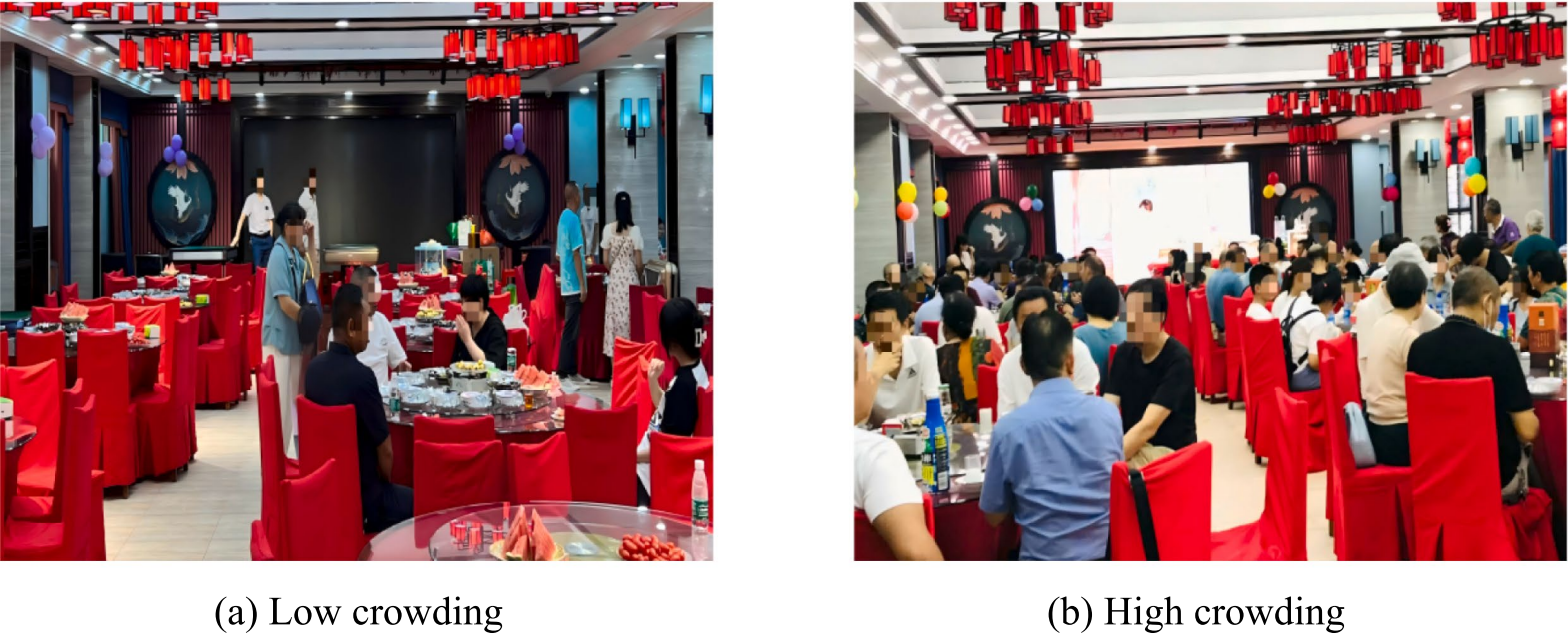
Crowding levels in the field experiment. **(a)** Low crowding. **(b)** High crowding.

We needed to determine the levels of crowding in the field experiment. The formal experiment was conducted in a Chinese restaurant. Following the crowding criteria proposed by Puzakova and Kwak (37), we defined the dining hall as a high crowding condition when more than 118 customers were present and the average personal space was less than 4.5 square feet. We defined a low crowding condition when fewer than 53 customers were present and the average personal space exceeded 10 square feet (see Figure 2). We randomly selected 30 customers in each condition and asked them about their perceived crowding (“How crowded do you feel? 1 = not crowded at all, 7 = very crowded”).

The results identified that consumers in the low crowding condition perceived significantly less crowding than those in the high crowding condition (*M*_low-crowding_ = 2.23, *SD* = 1.07; *M*_high-crowding_ = 6.27, *SD* = 0.64; *F* (1, 58) = 312.89, *p* < 0.001), confirming that the manipulation of crowding met the experimental requirements.

### 2.5 Method

This study used a 2 (crowding levels: low vs. high) × 3 (information-based intervention types: feedback-based interventions vs. prompt-type interventions vs. no interventions) between-participants design. We conducted a 30-day experiment in a Chinese restaurant in China. The experiment ran from 16 February to 20 March 2025. Due to staff holidays after the Chinese New Year, data collection was not performed for 3 days during this period. A total of 421 Tables (3,683 people) were recorded for the weight of leftover food.

The experiment was conducted in the restaurant lobby. The experimental area included 15 tables. Based on the results of Pretest 1, we prepared information-based intervention cards, including five prompt-type cards and five feedback-based cards. These cards were randomly placed at the center of 10 tables to ensure that consumers could easily see the content. The remaining five tables had no cards and served as the control group. Prior to the experiment, we trained the servers on how to weigh leftover food and record the results along with the corresponding information-based intervention type. Data obtained from this process in the experimental group were also included in the analysis. We also assigned on-site researchers to the restaurant. Their main tasks were to assess and record the real-time level of crowding based on the criteria used in Pretest 2. They also recorded the number of consumers and their gender at each table in the experimental area without using invasive procedures. After consumers left, following the procedure of Gao et al. (9), the servers responsible for cleaning placed the leftover food (excluding liquids) into a container on the scale and recorded its weight. In field experiments, this direct weighing method is often used to obtain first-hand data on consumer food waste (14). To ensure the data quality, we applied several procedures. First, all participating servers were selected through an initial screening. They were recommended by the headwaiter or manager and volunteered to participate. Second, servers were asked to write their names after each record. This allowed us to provide rewards and track the data quality. Third, each weighing and recording task was completed by two servers together. This approach improved table-cleaning efficiency and enabled mutual supervision. Considering that food waste might differ between lunch and dinner (38), the experiment was conducted during both lunch and dinner hours. The researchers periodically checked the experimental records and distributed rewards. Additionally, we rotated the position of the cards each week to avoid the potential impact of table location.

### 2.6 Results

We first conducted a 2 (crowding levels) × 3 (information-based intervention types) analysis of variance (ANOVA) on per capita food waste at the table without covariates, which revealed a significant interaction effect (*F* (2, 415) = 7.67, *p* = 0.001, *η*_p_^2^ = 0.036). Subsequently, we included the number of diners, dining time, number of male diners, and number of female diners as covariates, and the interaction effect remained significant (*F* (2, 411) = 8.20, *p* < 0.001, *η*_p_^2^ = 0.038).

Then, a simple effect analysis (see Table 1) showed that in low crowding conditions, the per capita leftover food at the table in the feedback-based group was lower than that in the prompt-type group (*M*_feedback_ = 158.11, *SD* = 8.33; *M*_prompt_ = 186.94, *SD* = 8.64; *p* = 0.048) and the control group (*M*_control_ = 217.03, *SD* = 8.75; *p* < 0.001), and the per capita leftover food at the table in the prompt-type group was also lower than that in the control group (*p* = 0.045). In contrast, in high crowding conditions, the per capita leftover food at the table in the prompt-type group was lower than that in the feedback-based group (*M*_prompt_ = 167.84, *SD* = 8.33; *M*_feedback_ = 205.34, *SD* = 8.35; *p* = 0.004) and the control group (*M*_control_ = 220.45, *SD* = 8.81; *p* < 0.001). However, the difference in per capita leftover food between the feedback-based group and the control group was not significant (*p* = 0.640), providing preliminary support for H1a and H1b. Additionally, there was no significant difference in per capita leftover food waste across different crowding levels (*F* (1, 129) = 0.208, *p* = 0.649).

**Table 1:** Results of the simple effect analysis (Study 1).

CL	IIT (I)	N	IIT (J)	*MD* (I-J)	*SD*	*p*	95%*CI*	
							*LLCI*	*ULCI*
High	No intervention	66	Feedback-based intervention	58.92^***^	12.08	<0.001	29.895	87.952
			Prompt-type intervention	30.10^*^	12.34	0.045	0.434	59.760
	Feedback-based	72	No intervention	−58.92^***^	12.08	<0.001	−87.952	−29.895
			Prompt-type intervention	−28.83^*^	11.94	0.048	−57.518	−0.135
	Prompt-type	72	No intervention	−30.10^*^	12.34	0.045	−59.760	−0.434
			Feedback-based intervention	28.83^*^	11.94	0.048	0.135	57.518
Low	No intervention	65	Feedback-based intervention	15.10	12.12	0.640	−14.026	44.229
			Prompt-type intervention	52.61^***^	12.07	<0.001	23.582	81.631
	Feedback-based	73	No intervention	−15.10	12.12	0.640	−44.229	14.026
			Prompt-type intervention	37.51^**^	11.70	0.004	9.387	65.623
	Prompt-type	73	No intervention	−52.61^***^	12.07	<0.001	−81.631	−23.582
			Feedback-based intervention	−37.51^**^	11.70	0.004	−65.623	−9.387

CL, crowding levels; IIT, information-based intervention type. The statistical significance: *p < 0.05; **p < 0.01; ***p < 0.001.

### 2.7 Discussion

Study 1 confirmed that, in low crowding conditions, although prompt-type interventions can somewhat reduce consumer leftover food compared with no interventions, the feedback-based interventions further reduced 15.4% of food waste. In high crowding conditions, prompt-type interventions reduced consumer food waste by an additional 18.2% compared with feedback-based interventions, while the effect of the feedback-based interventions was not significant. These findings provide preliminary support for the matching effect between crowding and the information-based intervention types. However, in the field experiment, it was difficult to explore the underlying mechanism in detail. Therefore, we need to conduct laboratory experiments to provide additional evidence.

### 2.8 Study 2

Study 2 was a laboratory experiment aimed at further testing the matching effect between crowding and information-based intervention types on consumer food waste and determining whether information processing fluency mediates this effect. When consumers face the possibility of leaving food uneaten in restaurants, attempting to avoid food waste by overeating may harm health (9). As a result, packing leftovers is considered one of the most effective ways to reduce consumer food waste (6). Yu et al. (15) demonstrated that packing leftovers has a significant impact on consumer food waste. Therefore, prior studies have treated packing leftovers as a proxy variable for consumer food waste (39). Based on this reasoning, following the approach of Gao et al. (9), we assessed consumers’ food waste intention by asking whether they were willing to take leftovers home.

Considering that crowded situations may trigger self-focus (40), which, in turn, may lead consumers to respond more positively to prompt-type interventions due to the need to maintain their self-image, we measured impression management motivation as an alternative explanation. Finally, prior research has indicated that factors such as gender (8), age (41), education level (24), income (42), and frequency of dining out (38) may influence food waste intentions and behaviors. Participants were asked to provide this information as control variables.

### 2.9 Method

The study followed a 2 (crowding levels: low vs. high) × 2 (information-based intervention types: feedback-based interventions vs. prompt-type interventions) between-participants design. We recruited 360 participants through the Credamo platform, and 351 participants passed the attention check (57.0% of female; *M*_age_ = 29.06, *SD* = 6.92).

First, we manipulated participants’ perceived crowding based on Hock and Bagchi (20). Participants were shown pictures of restaurant interiors with varying levels of crowding. The pictures were taken at the restaurant where the field experiment was conducted in Study 1. To protect privacy, all faces of individuals in the pictures have been blurred. Participants were required to imagine dining in the environment depicted in the picture and write down their feelings in terms of the number of people, interpersonal distance, and available space (a minimum of 15 characters). They then reported their perceived crowding (How crowded would you feel in this situation? 1 = not crowded at all, 7 = very crowded). We then informed the participants that there was a card placed on the table at the restaurant. To improve the robustness of the findings, we made limited adjustments to the stimulus content while keeping the font, background, borders, and other design elements unchanged. Specifically, the prompt-type information in Study 1 used rhyming language in Chinese. This feature may have influenced participants’ impressions and attitudes. Therefore, in Study 2, we made the prompt-type information more direct and removed the rhyming structure. The card used in the prompt-type intervention group stated, “Pack up the leftover food to take away.” Similarly, following Whitehair et al. (10), we adjusted the feedback-based information to report food waste on a per capita, per meal basis. The card used in the feedback-based intervention group stated, “On average, each consumer wastes 93 g of food each meal.” After this, following Gao et al. (9), we asked the participants what they would do with any leftover food after finishing their meal by three items: “I would throw away the remaining food” (1 = strongly disagree, 7 = strongly agree; reverse-coded), “I would save the remaining food to eat” (1 = strongly disagree, 7 = strongly agree), and “Think more precisely about the scenario and your potential actions, what would you do? ““(1 = I’d definitely throw away what’s left of the meal, 7 = I’d definitely save the leftovers to eat; *α* = 0.91). Next, we measured participants’ information processing fluency. Afterward, we measured participants’ information processing fluency. It is important to note that information processing fluency is relatively vague, abstract and difficult to accurately perceive (29). As a result, directly asking participants whether they feel fluent often yields suboptimal results (43). Therefore, scholars generally turn to designing measurement items based on the outcomes of fluency, such as improved evaluations of the information, to capture processing fluency indirectly (44). Including these items helps reduce measurement error (29), but it can also inevitably overlap with variables such as clarity or perceived information quality. Since all participants were Chinese consumers, we adopted a well-established information processing fluency scale developed by Jin and Zhu (28). It includes five items (e.g., “After reading the information on the card, I can imagine the related scene,” 1 = strongly disagree, 7 = strongly agree; α = 0.80). Jin and Zhu (28) demonstrated that these items show sufficient internal consistency and face validity for measuring information processing fluency among Chinese consumers. Following Peloza et al. (45), we measured impression management motivation as a potential alternative explanation by three items (e.g., “I want to make myself look good to others”; 1 = strongly disagree, 7 = strongly agree; α = 0.87). Subsequently, participants reported their gender, age, education level, income, and frequency of dining out. Finally, we conducted a manipulation check of the stimuli using the same procedure as Pretest 1 in Study 1. Participants were provided with the definitions of prompt-type interventions and feedback-based interventions. They were then asked to identify which type the card they had just seen belonged to (see Table 2).

**Table 2:** Constructs and measurements in Study 2.

Construct	Item	Description	Source
Willingness to pack leftovers	WPL 1	I would throw away the remaining food.	Gao et al. (9)
	WPL 2	I would save the remaining food to eat.	
	WPL 3	Think more precisely about the scenario and your potential actions. What would you do?	
Information processing fluency	IPF 1	The information on the card is easy to understand.	Jin and Zhu (28)
	IPF 2	After reading the information on the card, I can imagine the related scene.	
	IPF 3	The structure of the information on the card is reasonable	
	IPF 4	The information on the card is well organized.	
	IPF 5	The meaning of the information on the card is clear and logical.	
Impression management motivation	IMM 1	I want to present myself in a positive way to others.	Peloza et al. (45)
	IMM 2	I want to make a positive impression on others.	
	IMM 3	I want to make myself look good to others.	

### 2.10 Results

#### 2.10.1 Manipulation check

We found that participants’ perceived crowding is significantly higher in the high crowding group (*M*_low-crowding_ = 2.70, *SD* = 1.19; *M*_high-crowding_ = 5.91, *SD* = 1.00; *F* (1, 349) = 751.74, *p* < 0.001). Participants in the feedback-based intervention group significantly believed that the card they viewed belonged to a feedback-based intervention (*M*_feedback_ = 5.05, *SD* = 1.88; *M*_prompt_ = 3.25, *SD* = 2.07; *F* (1, 349) = 75.57, *p* < 0.001). Participants in the prompt-type intervention group significantly believed that the card they viewed belonged to a prompt-type intervention (*M*_feedback_ = 1.86, *SD* = 1.24; *M*_prompt_ = 6.39, *SD* = 0.83; *F* (1, 356) = 1647.93, *p* < 0.001). The difference between the two groups in terms of the esthetic appeal of the card was not significant (*M*_feedback_ = 5.22, *SD* = 1.06; *M*_prompt_ = 5.14, *SD* = 1.13; *F* (1, 349) = 0.42, *p* = 0.518).

#### 2.10.2 Interaction effect analyses

We conducted a 2 (crowding levels) × 2 (information-based intervention types) ANOVA on willingness to pack leftovers without covariates, which revealed that the main effects of crowding (*F* (1, 347) = 0.75, *p* = 0.388) or information intervention (*F* (1, 347) = 0.004, *p* = 0.948) were not significant, but the interaction effect between crowding and information-based intervention types was significant (*F* (1, 347) = 13.57, *p* < 0.001, *η*_p_^2^ = 0.038). Subsequently, we included gender, age, education level, income, and dining frequency as covariates. The results identified that the main effects of crowding (*F* (1, 342) = 0.34, *p* = 0.559) or information intervention (*F* (1, 342) = 0.01, *p* = 0.952) were not significant, but the interaction effect between crowding and information-based intervention types was significant (*F* (1, 342) = 15.79, *p* < 0.001, *η*_p_^2^ = 0.044).

**Figure 3:**
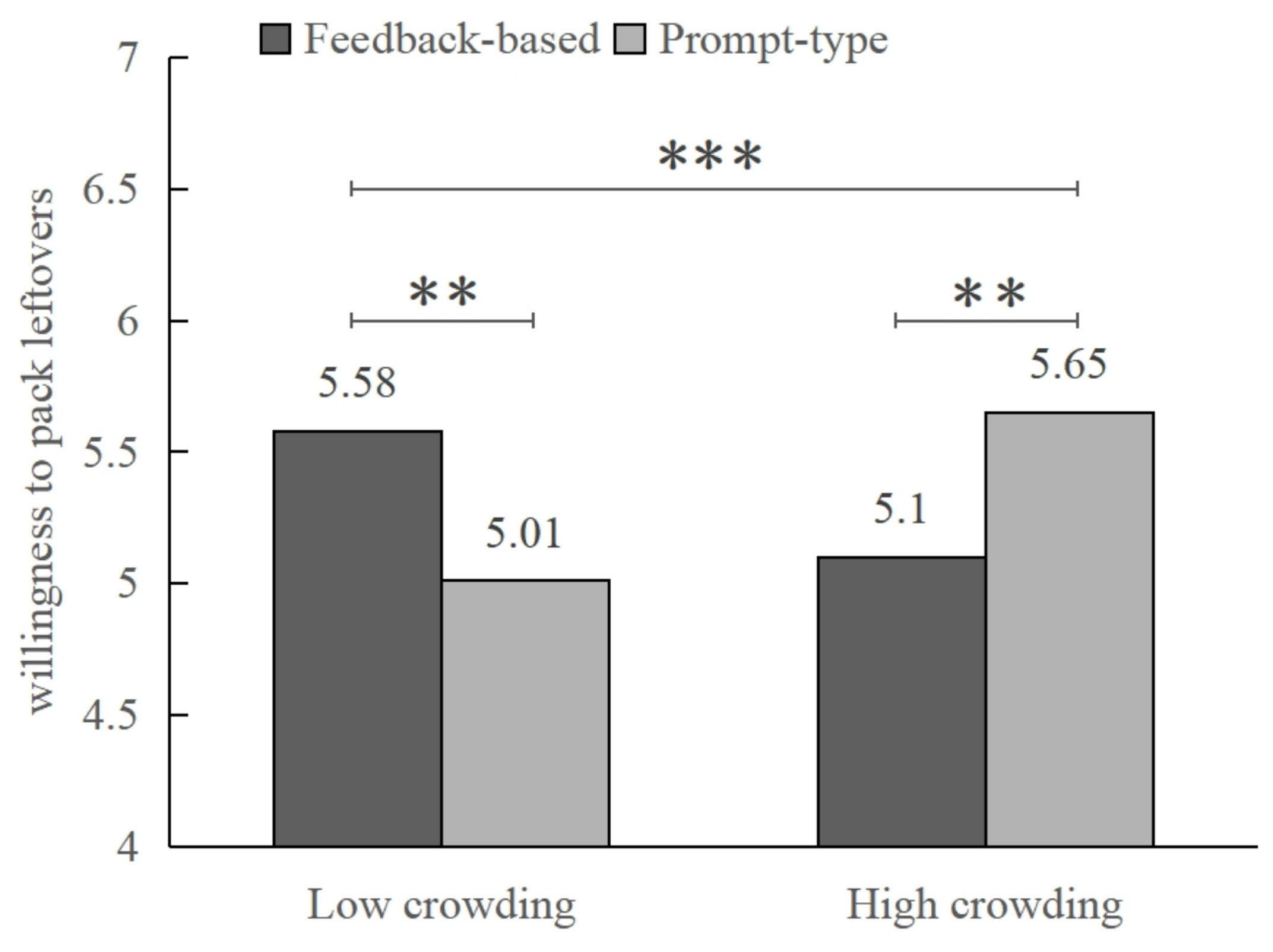
Interaction effect of crowding and information-based interventions on willingness to pack leftovers (Study 2). **p < 0.01; ***p < 0.001.

Then, a simple effect analysis revealed that, in low crowding conditions, consumers who received feedback-based interventions showed a higher willingness to pack leftovers than those who received prompt-type interventions (*M*_feedback_ = 5.58, *SD* = 0.14; *M*_prompt_ = 5.01, *SD* = 0.14; *F* (1, 342) = 8.09, *p* = 0.005, *η*_p_^2^ = 0.023). In contrast, in high crowding conditions, prompt-type interventions were more effective than feedback-based interventions in increasing consumers’ willingness to pack leftovers (*M*_feedback_ = 5.10, *SD* = 0.14; *M*_prompt_ = 5.65, *SD* = 0.14; *F* (1, 342) = 7.59, *p* = 0.006, *η*_p_^2^ = 0.022; see Figure 3), which further validated H1a and H1b.

#### 2.10.3 Mediation analyses

First, we conducted a 2 (crowding levels) × 2 (information-based intervention types) ANOVA on information processing fluency, gender, age, education level, income, and dining frequency as covariates. The results showed a significant interaction between crowding and information-based intervention types on information processing fluency (*F* (1, 342) = 43.74, *p* < 0.001, *η*_p_^2^ = 0.113).

**Figure 4:**
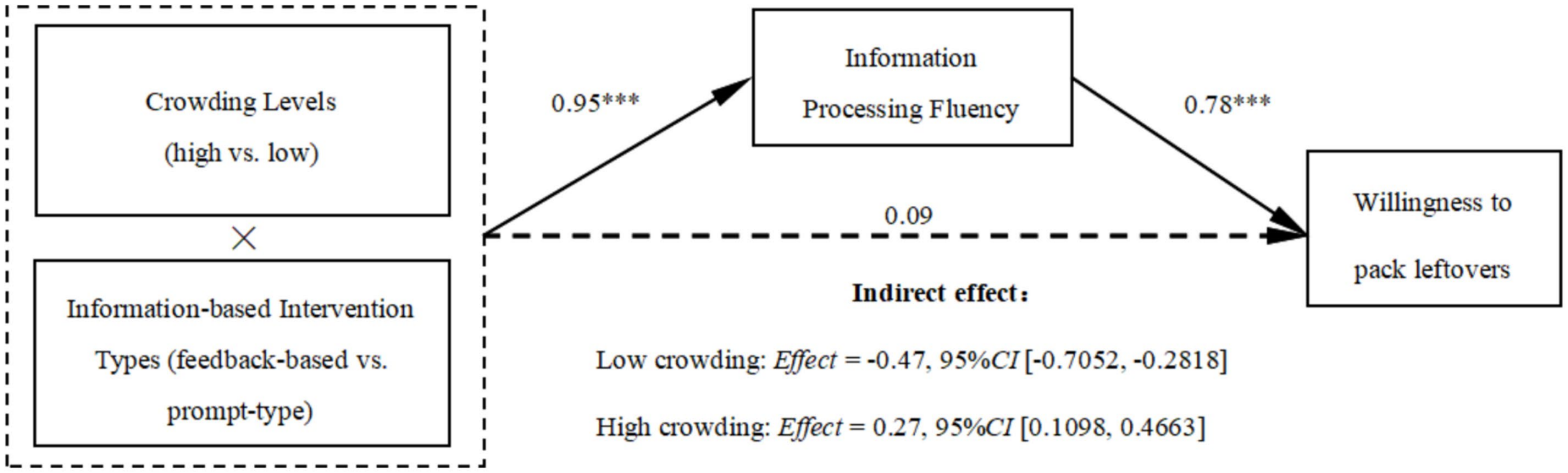
Results of mediation analyses (Study 2). ***p < 0.001.

Subsequently, a bootstrap analysis (Model 7, 5,000 bootstraps, PROCESS; low crowding = 0, high crowding = 1; feedback-based = 0, prompt-type = 1) was performed, revealing a significant mediation effect of information processing fluency (*Index* = 0.74*, SE* = 0.17, *LLCI* = 0.4495, *ULCI* = 1.0939). Specifically, in low crowding conditions, the mediation effect of information processing fluency was significantly negative (*Effect* = −0.47, *SE* = 0.11, *LLCI* = −0.7052, *ULCI* = −0.2818), indicating that participants perceived higher information processing fluency with feedback-based interventions, leading to a higher willingness to pack leftovers. In high crowding conditions, the mediation effect of information processing fluency was significantly positive (*Effect* = 0.27, *SE* = 0.09, *LLCI* = 0.1098, *ULCI* = 0.4663), indicating that participants perceived higher information processing fluency with prompt-type interventions, resulting in a higher willingness to pack leftovers (see Figure 4), supporting H2.

Additionally, we conducted a similar bootstrap analysis to test whether impression management motivation mediated the effect and found that this hypothesis was not supported (*Index* = −0.0053, *SE =* 0.03, *LLCI* = −0.0591, *ULCI* = 0.0741), thus ruling out its potential as an alternative explanation.

### 2.11 Discussion

Study 2 confirmed that crowding and the information-based intervention types interact to influence consumers’ willingness to pack leftovers, providing further support for H1a and H1b. Additionally, Study 2 found that information processing fluency mediates this effect and ruled out impression management motivation as an alternative explanation. We consider that there may be a potential gap between willingness and actual behavior, and previous studies suggest that over-ordering is another key behavioral factor contributing to food waste (46). Therefore, Study 3 will directly measure the matching effect between crowding and information-based intervention types on consumers’ ordering behavior to test the hypotheses.

## 3 Study 3

In Study 3, we changed the stimuli and the measurement methods of the dependent variable. The aim was to recheck the interaction effect between crowding and information-based intervention types and to determine whether information processing fluency mediates this effect. Consumer food waste in restaurants is essentially caused by ordering more food than consumers can eat (47). As a result, over-ordering is widely regarded as one of the main causes of food waste (41). This view has motivated scholars to develop measurement approaches based on ordering behavior. For example, Coşkun and Özbük (48) developed an Intention to Reduce Food Waste (INT) scale based on ordering decisions. Xu et al. (26) measured consumer food waste using a simulated ordering task. Over-ordering is often driven by a lack of awareness about portion sizes or low food-saving consciousness (46). This suggests that when menus provide clear portion size information, consumers who wish to avoid food waste are more likely to control their ordering weight (49). Building on this, in Study 3, we designed a simulated ordering task to directly measure consumers’ ordering behavior to assess potential food waste.

### 3.1 Method

The study followed a 2 (crowding levels: low vs. high) × 2 (information-based intervention types: feedback-based interventions vs. prompt-type interventions) between-participants design. We recruited 360 participants through the Credamo platform, and 352 participants passed the attention check (56.3% of female; *M*_age_ = 28.27, *SD* = 7.95).

**Figure 5:**
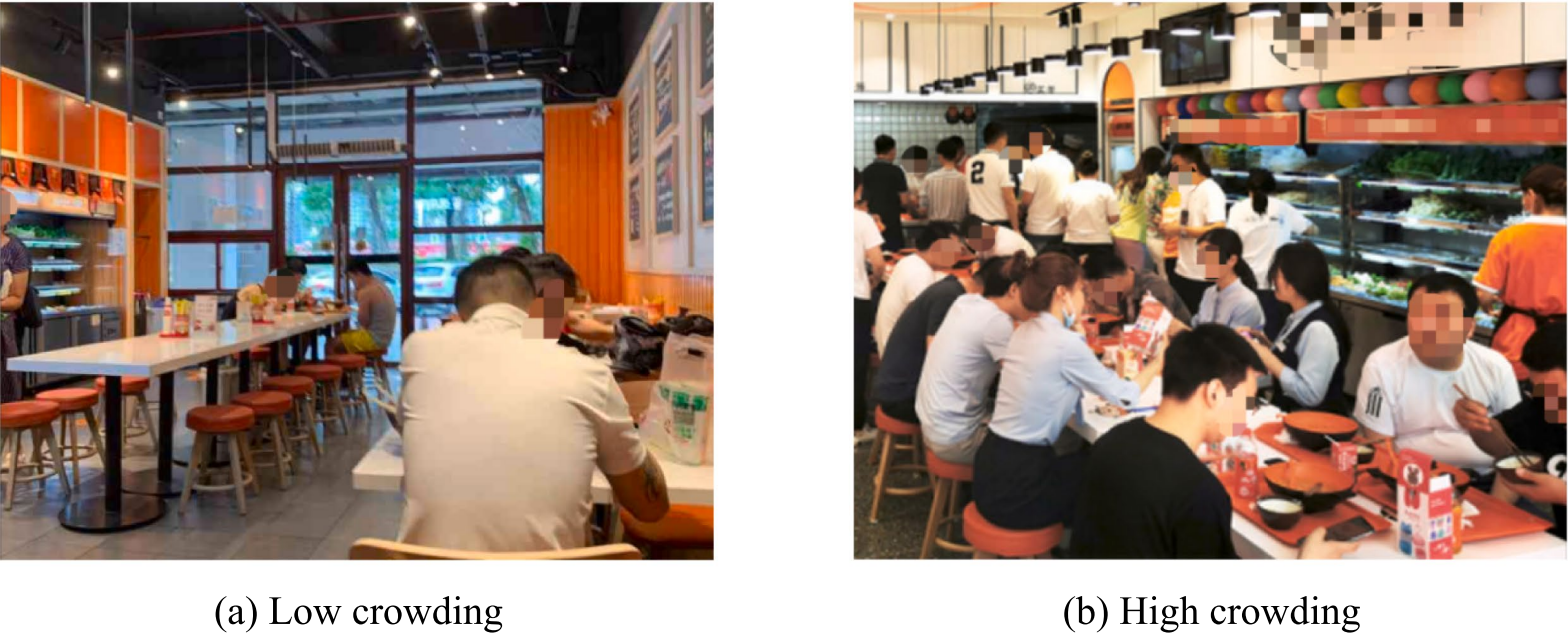
Manipulations of crowding levels in Study 3. **(a)** Low crowding. **(b)** High crowding.

Similar to Study 2, we showed participants an interior picture of a spicy hotpot restaurant (one of the most popular Chinese dining types in which consumers select multiple food items in small portions from a menu, and the cooks then cook all selected items together), where the low crowding group saw a picture with approximately 7 people, and the high crowding group saw a picture with 30 people. The faces of the customers in the pictures were blurred (Figure 5). Participants were required to imagine dining as shown in the picture and describe their feelings based on factors such as the number of people around them, as well as report their perceived crowding. Participants were then informed that there was a card placed on the table at the restaurant. Participants were then informed that there was a card placed on the table at the restaurant. To improve the robustness of the findings, we made limited adjustments to the stimulus content. Specifically, Study 3 focused on consumers’ over-ordering behavior. Therefore, we revised the prompt-type information to encourage appropriate ordering. The prompt-type intervention card stated, “Order appropriately, start with me.” In addition, Studies 1 and 2 used individual-level feedback-based information. Therefore, following Xu et al. (26), we designed group-level feedback-based information in Study 3, and the feedback-based intervention card stated, “Chinese consumers waste 18 million tons of food in restaurants every year.” To ensure comparability across the results, the stimuli in Study 3 used the same font, background, border design, and wording style as those in Studies 1 and 2. Afterward, participants were provided with an electronic spicy hotpot menu. The menu included a wide range of food options. Each item was labeled with its weight and price (e.g., mushrooms, 50 g, 2.28 RMB). Participants were asked to imagine that they were ordering food and to select the food items they wished to purchase. The total weight of all selected items was used to measure ordering behavior. Then, participants evaluated the reasonableness of the menu design (“Do you think the design of this menu is reasonable? 1 = very unreasonable, 7 = very reasonable”). Subsequently, the same scale used in Study 2 was used to measure participants’ information processing fluency (*α* = 0.74), and demographic information was collected. Finally, we conducted a manipulation check of the stimuli using the same procedure as Pretest 1 in Study 1. Participants were provided with the definitions of prompt-type interventions and feedback-based interventions. They were then asked to identify which type the card they had just seen belonged to.

### 3.2 Results

#### 3.2.1 Manipulation check

Participants’ perceived crowding is significantly higher in the high crowding group (*M*_low-crowding_ = 2.58, *SD* = 1.26; *M*_high-crowding_ = 5.79, *SD* = 1.07; *F* (1, 350) = 669.04, *p* < 0.001). Participants in the feedback-based intervention group significantly believed that the card they viewed belonged to a feedback-based intervention (*M*_feedback_ = 5.39, *SD* = 1.66; *M*_prompt_ = 2.92, *SD* = 1.85; *F* (1, 352) = 176.01, *p* < 0.001). Participants in the prompt-type intervention group significantly believed that the card they viewed belonged to a prompt-type intervention (*M*_feedback_ = 2.03, *SD* = 1.35; *M*_prompt_ = 5.89, *SD* = 1.11; *F* (1, 348) = 854.40, *p* < 0.001). There was no significant difference in the ratings for the esthetic appeal of the cards (*M*_feedback_ = 5.43, *SD* = 0.91; *M*_prompt_ = 5.46, *SD* = 0.90; *F* (1, 350) = 0.12, *p* = 0.728) and the evaluation of the reasonableness of the menu design (*M*_feedback_ = 5.54, *SD* = 1.01; *M*_prompt_ = 5.66, *SD* = 1.05; *F* (1, 350) = 1.09, *p* = 0.287), indicating that the design of the stimuli met the experimental requirements.

#### 3.2.2 Interaction effect analyses

We conducted a 2 (crowding levels) × 2 (information-based intervention types) ANOVA on the ordering weight without covariates, which revealed that the main effects of crowding (*F* (1, 348) = 1.04, *p* = 0.308) or information intervention (*F* (1, 348) = 0.27, *p* = 0.601) were not significant, but the interaction effect between crowding and information-based intervention types was significant (*F* (1, 348) = 17.44, *p* < 0.001, *η*_p_^2^ = 0.048). Subsequently, we included gender, age, education level, income, and dining frequency as covariates. The results revealed that the main effect of crowding (*F* (1, 343) = 0.99, *p* = 0.321) or information-based intervention types (*F* (1, 343) = 0.19, *p* = 0.664) was not significant, but the interaction effect between crowding and information-based intervention types was significant (*F* (1, 343) = 19.27, *p* < 0.001, *η*_p_^2^ = 0.053).

**Figure 6:**
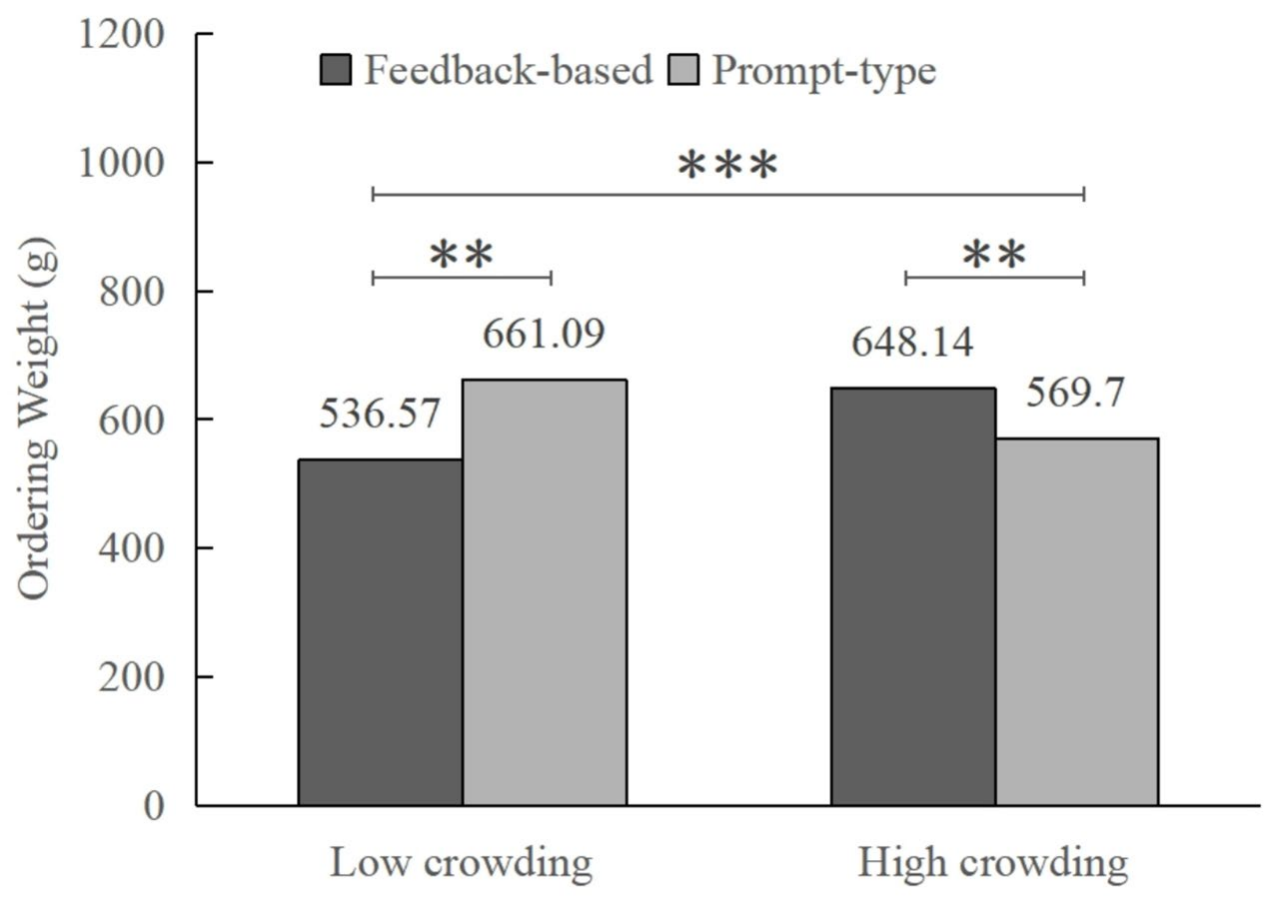
Interaction effect of crowding and information-based interventions on ordering weight (Study 3). **p < 0.01; ***p < 0.001.

Further simple effect analysis revealed that in low crowding conditions, participants who received feedback-based interventions ordered significantly less than those who received prompt-type interventions (*M*_feedback_ = 536.57, *SD* = 23.12; *M*_prompt_ = 648.14, *SD* = 23.57; *F* (1, 343) = 8.09, *p* = 0.001, *η*_p_^2^ = 0.032). In contrast, in high crowding conditions, prompt-type interventions were more effective than feedback-based interventions in reducing consumers’ over-ordering behavior (*M*_feedback_ = 661.09, *SD* = 23.07; *M*_prompt_ = 569.70, *SD* = 22.87; *F* (1, 343) = 7.91, *p* = 0.005, *η*_p_^2^ = 0.023; see Figure 6), which provides further evidence for H1a and H1b.

#### 3.2.3 Mediation analyses

A 2 (crowding levels) × 2 (information-based intervention types) ANOVA was conducted on information processing fluency. We found that the interaction effect between crowding and information-based intervention types on information processing fluency was significant (*F* (1, 343) = 27.79, *p* < 0.001, *η*_p_^2^ = 0.075).

**Figure 7:**
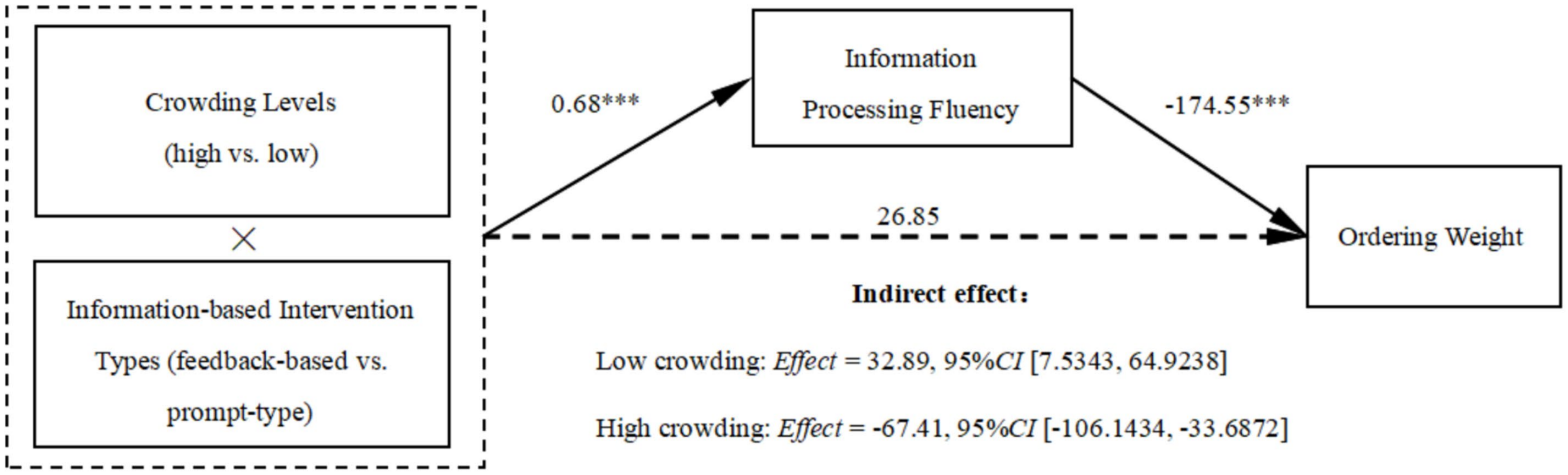
Results of mediation analyses (Study 3). ***p < 0.001.

Subsequently, we conducted a bootstrap analysis (Model 7, 5,000 bootstraps, PROCESS; low crowding = 0, high crowding = 1; feedback-based = 0, prompt-type = 1). The results indicate that the mediating effect of information processing fluency is significant (*Index* = −100.30, *SE* = 26.69, *LLCI* = −158.4871, *ULCI* = −51.9263). Specifically, in low crowding conditions, the mediation effect of information processing fluency was significantly negative (*Effect* = 32.89, *SE* = 14.53, *LLCI* = 7.5343, *ULCI* = 64.9238), indicating that participants in the feedback-based intervention group perceived higher information processing fluency, which led to a reduction in over-ordering behavior. In high crowding conditions, participants who received prompt-type interventions perceived higher information processing fluency than those who received feedback-based interventions, which led them to order significantly less (*Effect* = −67.41, *SE* = 18.45, *LLCI* = -106.1434, *ULCI* = -33.6872; see Figure 7), supporting H2.

### 3.3 Discussion

In Study 3, we replaced the stimuli and validated the matching effect between crowding and information-based intervention types on consumers’ over-ordering behavior through a simulated ordering task. It also demonstrates that information processing fluency mediates this effect, further strengthening the robustness of the study’s conclusions.

## 4 General discussions and implications

Information-based interventions are considered an important measure in addressing food waste practices. However, evaluations of their effectiveness remain limited, and conclusions are often conflicting (14). In particular, scholars have reported opposite conclusions regarding the effectiveness of two common types of information-based interventions in anti-food waste campaigns: prompt-type interventions and feedback-based interventions (10, 26). This situation suggests the presence of unidentified moderating factors influencing the effect of information-based interventions on consumer food waste (17).

Based on a review of the literature, we observe that scholars in consumer behavior have emphasized the strong influence of environmental factors in consumption settings (18). However, prior research on anti-food waste information has largely focused on the interaction between intervention materials and consumer behavior (7, 13). It has paid limited attention to the potential role of environmental factors. This omission may help explain why existing evaluations of anti-food waste information show inconsistent results. To address this gap, this study incorporates crowding, a common environmental factor in restaurant settings. Drawing on processing fluency theory, we develop a matching model between information-based intervention type and crowding level. Specifically, we propose that under low crowding conditions, feedback-based interventions are more effective than prompt-type interventions in reducing consumer food waste. In contrast, under high crowding conditions, prompt-type interventions are more effective than feedback-based interventions. Information processing fluency serves as the mediating mechanism underlying this matching effect.

We conducted three experiments to test the hypotheses. In Study 1, we placed information-based intervention cards on the tables of a Chinese restaurant and evaluated the effects of these cards on the weight of leftover food under different crowding levels. The results showed that when the restaurant was less crowded, both information-based intervention types significantly reduced consumer food waste compared with no interventions. Moreover, the feedback-based interventions reduced the weight of leftover food by an additional 15.4% compared with the prompt-type interventions. This result replicated the findings of Liao et al. (25) and Xu et al. (26). However, when the restaurant was more crowded, the simple and direct prompt-type interventions reduced the weight of leftover food by an additional 18.2% compared with the feedback-based interventions, while the feedback-based interventions did not show a significant effect compared with no interventions. This result was consistent with the findings of Whitehair et al. (10). Based on this, in Studies 2 and 3, we followed Hock and Bagchi (20) and manipulated participants’ perceived crowding using images. We then measured their willingness to pack leftovers and over-ordering behavior after they viewed different information-based intervention cards. The results replicated the findings of Study 1 and further showed that information processing fluency mediated this matching effect.

### 4.1 Theoretical contribution

First, this study develops a matching model between the environmental factors and information-based intervention types to provide a scientific explanation for the ongoing debate on the effectiveness of information-based interventions in restaurants. Prior research has not reached a consensus on which type of information intervention is more effective in reducing consumer food waste. This study is the first to show that feedback-based interventions are more effective in non-crowded environments, whereas prompt-type interventions perform better in crowded environments. This finding highlights the important moderating role of environmental factors in intervention effectiveness. It also encourages future research to move beyond static comparisons of information-based intervention types and more fully consider how to maximize the effectiveness of information interventions.

Second, this study reveals that information processing fluency is the key psychological mechanism underlying the matching effect between crowding and information-based intervention types. In doing so, it fills an important gap in the anti-food waste literature. Although Whitehair et al. (10) noted that different types of information-based interventions require different processing routes, they did not examine how consumers select these routes or identify the psychological mechanisms behind this effect. Our findings show that crowding influences consumers’ information processing routes and interacts with information-based intervention types. Information processing fluency serves as the key pathway through which this matching effect influences consumer food waste. These results extend prior research, deepen understanding of how information-based interventions shape consumer behavior, and broaden the application of processing fluency theory.

Third, this study advances crowding research by challenging the dominant view that crowding only has negative effects on consumer outcomes. Previous studies have mainly emphasized the negative impact of crowding. For example, crowding reduces consumer satisfaction (50), increases social withdrawal (37), and impairs rational thinking (20). In contrast, this study identifies a positive effect of crowding. High crowding levels can enhance consumers’ processing fluency for prompt information, which improves the effectiveness of prompt-based interventions. This finding offers a new perspective on the potential positive role of crowding in consumption settings.

### 4.2 Practical implications

Dining in restaurants has led to a rapidly growing amount of food waste (6). Information-based interventions are considered an important measure for mitigating consumer food waste and have been widely implemented in restaurants across many countries (7). In practice, many marketers assume that mass-producing information-based intervention materials (e.g., labels, cards, or posters), and placing them in all restaurants will effectively reduce food waste. This study challenges this assumption. The findings of this study show that environmental factors in restaurants influence the effectiveness of information-based interventions. This means that using intervention information without considering context is unlikely to achieve the desired outcomes. Based on these insights, this study offers practical guidance for marketers on how to apply information-based interventions more effectively to reduce food waste.

First, crowding has become a common environmental factor in restaurants (19). The persuasiveness of different types of information-based interventions varies across crowding levels. Under low crowding conditions, feedback-based interventions are more effective than prompt-type interventions in reducing consumer food waste. In contrast, under high crowding conditions, prompt-type interventions perform better than feedback-based interventions. Therefore, marketers must consider the potential impact of crowding levels when using information-based interventions to encourage consumers to reduce food waste. For example, from a spatial perspective, upscale restaurants typically offer a more spacious and comfortable dining environment with lower crowding levels, where marketers can more effectively use feedback-based interventions. In contrast, fast-food restaurants are usually more crowded and may benefit from using simple and direct prompt-type interventions. From a temporal perspective, during peak dining times such as weekends or holidays, marketers can implement more prompt-type interventions in restaurants, while during off-peak hours, it may be beneficial to use more feedback-based interventions to curb consumer food waste.

Second, this study not only identifies a matching effect between crowding level and information-based intervention type but also shows that information processing fluency is a key mechanism underlying this effect. This finding provides a practical guideline for marketers when evaluating intervention effectiveness: effective information-based interventions should aim to ensure high processing fluency for consumers. Prior research showed that factors such as information exposure frequency (51) or font design (52) can also influence processing fluency. Thus, intervention information should be presented using easy-to-read fonts, and the amount of sustainability information in restaurants should be increased to improve the overall effectiveness of the intervention.

### 4.3 Limitations and future research

First, the findings of this study are subject to certain boundary conditions. This research focuses on the interaction between human crowding, caused by the presence of a high density of individuals in a specific location, and types of information intervention. Prior studies suggested that spatial crowding and human crowding have different effects on consumers (37). For example, spatial crowding does not disrupt consumers’ cognitive resources as strongly as human crowding does (20). As a result, the findings of this study may not apply to situations of spatial crowding. Second, although we controlled for personal factors such as gender and age, other variables such as consumer arousal, stress, time pressure, or service quality may also influence consumer food waste behavior (46). Future studies could design more comprehensive experiments by including these factors as control variables. Third, the information-based interventions in the experiments were presented in the form of static cards. As a result, the feedback-based information lacked sufficient personalization and timeliness. With the development and wide use of smart devices, food waste in restaurants may be tracked and reported to consumers in real time. This may strengthen the effects of feedback-based interventions (53). Therefore, whether the conclusions of this study apply to this situation requires further exploration. Moreover, the feedback-based information in this study reported the Chinese Academy of Sciences as the data source. Although we reduced the font size of this information as much as possible, it may still have influenced the effectiveness of the intervention. Future research could examine whether the presence of a source, or different types of sources (e.g., government vs. non-government organizations), affects the effectiveness of information-based interventions. In addition, although this study used established scales that have been developed and validated by previous research to measure information processing fluency, some of the items that indirectly assess processing fluency based on its outcomes overlap with concepts such as clarity and perceived information quality, which may somewhat reduce the discriminant validity of the results. Future research could consider developing measurement tools that better align with the definition of information processing fluency and further test the conclusions of this study. Finally, although this study confirms that information-based interventions aligned with environmental factors can effectively increase consumers’ willingness to pack leftovers, several negative factors in real-world settings may hinder consumers’ packing decisions. For example, packing leftovers often involves an additional cost. In addition, prior research showed that in some countries, consumers view packing leftovers as a violation of social norms (54) or consider requesting a takeaway container from service staff to be embarrassing (55). These factors may limit the translation of willingness into actual behavior. Future research may consider incorporating these potential constraints to enhance the practical relevance of the findings.

## Data Availability

The raw data supporting the conclusions of this article will be made available by the authors, without undue reservation.
